# Evaluation of Liver Fibrosis Change After DAA-induced Cure of Hepatitis C in Participants With and Without HIV: ACTG A5320 Viral Hepatitis C Infection Long-term Cohort Study (VHICS)

**DOI:** 10.1093/ofid/ofaf804

**Published:** 2026-01-14

**Authors:** Marion G Peters, Minhee Kang, Robert Murphy, William Rosenberg, David L Wyles

**Affiliations:** Department of Medicine, Northwestern University, Chicago, Illinois, USA; Center for Biostatistics in AIDS Research in the Department of Biostatistics, Harvard T. H. Chan School of Public Health, Boston, Massachusetts, USA; Department of Medicine, Northwestern University, Chicago, Illinois, USA; UCL Institute for Liver and Digestive Health, The Royal Free London NHS Foundation Trust, London, UK; Department of Medicine, Denver Health Medical Center, Denver, Colorado, USA

**Keywords:** cohort study, ELF fibrosis marker, fibrosis, follow up of HCV cure, HIV

## Abstract

Change in liver fibrosis was studied over 5 years after cure of hepatitis C (HCV) in participants with and without HIV from the Viral Hepatitis C Infection Long-Term Cohort Study (VHICS). Markers of liver fibrosis included aspartate aminotransferase to platelet ratio (APRI), fibrosis 4 index (FIB-4), and a direct measure of extracellular matrix, enhanced liver fibrosis (ELF). We evaluated 122 participants without HIV and 128 with HIV. At study entry, which occurred on average 30 weeks after antiviral completion, more participants had severe fibrosis by ELF (21%) than FIB-4 (7%) or APRI (1%). ELF scores were not available before entry into VHICS. The proportions of participants in predefined ELF categories were similar between the 2 groups at study entry and over time. Advanced fibrosis by ELF did not decrease over time. Clinical events were observed in 44 (12%): 29 HCV/HIV and 15 HCV participants. HCV/HIV participants had a 1.95 times higher risk of developing a clinical event, compared to HCV. A lower entry ELF score was numerically associated with a lower risk of a clinical event. There was an association between VHICS entry ELF and time to first targeted liver diagnosis or all-cause death (hazard ratio; 95% CI, 0.268 [.094-.763], *P* = .014). In conclusion, APRI and FIB-4 decreases occurred early after direct-acting antiretroviral therapy, likely from decreased necroinflammation. ELF identified participants who continued to have advanced liver fibrosis and was associated with development of liver outcomes and death. Studies after sustained virologic response should include longer term follow up to monitor for clinical events.

## Teaser

ELF fibrosis marker, studied over 5 years after SVR, identified HCV and HCV/HIV participants with continued advanced fibrosis at higher risk of development of adverse liver outcomes and death.

Assessment of liver fibrosis can be challenging after cure of hepatitis C virus (HCV) because many noninvasive markers of liver fibrosis incorporate tests of liver inflammation, such as aspartate aminotransferase (AST) and alanine aminotransferase (ALT). These tests, although readily available, are less sensitive and not specific compared to “direct markers” of fibrosis, such as components of liver matrix and mediators of matrix remodeling [1]. These latter components are incorporated in the enhanced liver fibrosis test (ELF), which uses hyaluronic acid, procollagen III amino terminal peptide, and tissue inhibitor of matrix metalloproteinase [2]. In addition to studying APRI and FIB-4, we chose ELF as it has been validated and shown to be superior to APRI and FIB-4 in people with HCV, without and with HIV, and in other chronic liver diseases; uses direct measures of extracellular matrix components as noted previously; and can be performed on stored serum [3, 4].

The A5320 Viral Hepatitis C Infection Long-term Cohort Study (VHICS) from ACTG (Advancing Clinical Therapeutics Globally, formerly AIDS Clinical Trials Group) followed participants who had achieved a sustained virologic response (SVR) after direct-acting antiviral (DAA)-based HCV treatment. The primary aim of this substudy was to assess long-term liver fibrosis evolution by ELF in participants following HCV cure. Liver fibrosis was measured by ELF and the more commonly used serum markers APRI and FIB-4 at study entry and semiannually up to 5 years. The secondary aims were to compare liver fibrosis in those with and without HIV and to evaluate the association between ELF at study entry and the development of clinical events during the study.

## METHODS

A5320 (VHICS) was a long-term prospective study that enrolled participants with and without HIV (HCV/HIV and HCV) within 1 year of completion of DAA-based HCV treatment. Study follow-up was up to 5 years and full details have been previously published [5]. This analysis included those participants with SVR who had at least 1 year of follow-up in VHICS. SVR was defined as HCV RNA less than the lower limit of quantification at least 12 weeks after treatment completion with no known subsequent HCV RNA ≥ lower limit of quantification. Five participants had recurrence of HCV: only the data before HCV recurrence were used in the postentry analyses. Study visits included clinical and laboratory assessments as detailed in the primary manuscript [5]. Homeostasis model assessment of insulin resistance (HOMA-IR) was measured to assess insulin resistance [6]. HOMA-IR >3 was considered evidence of insulin resistance.

Three serum markers of fibrosis were tested at week 0 (VHICS entry) and weeks 26 through 260 at half-yearly intervals. Data for APRI and FIB-4 were also available before DAA therapy. ELF (Siemens Healthcare Diagnostics, Tarrytown, New York) was assayed on an automated IMMUNO 1 immunoanalyzer (Siemens Medical Solutions Diagnostics, Tarrytown, New York) as described [4]. The ELF markers (hyaluronic acid, procollagen III amino terminal peptide, and tissue inhibitor of matrix metalloproteinase-1) were analyzed individually, and the results continually referred to a set of quality standards to ensure accurate analysis. Tests were performed according to the manufacturer's instructions at iQur Limited, London, UK. Each ELF score for fibrosis was reviewed in 3 ways: first, as a continuous measure; second, categorized as none to mild fibrosis (F0-1 < 7.7), moderate to advanced (F2: 7.7 to <9.8); advanced to severe (F3: 9.8 to <11.3) and cirrhosis (F4: ≥11.3); and third, dichotomized as below F3 fibrosis (<9.8) and advanced fibrosis (F3/4 ≥ 9.8). Our focus was on advanced fibrosis. Twenty-one participants (8%) had ELF data for all 10 scheduled weeks and most (77%) participants had ≥6 ELF scores. The indirect markers of liver fibrosis used were APRI [7] and FIB-4 [8]. Both APRI and FIB-4 were evaluated as continuous variable and dichotomized to severe fibrosis (no/yes) as defined by APRI ≤1.5 and >1.5 and FIB-4 ≤ 3.25 and >3.25. Changes from week 0 in APRI of 0.25, FIB-4 0.5, and ELF 0.5 were considered clinically significant [9].

Vibration-controlled transient elastography (VCTE) results were included where available and each was scored as a continuous measure at the time prior to DAA treatment initiation, and after VHICS entry at years 1 through 5. VCTE was also categorized as none to mild fibrosis (F0-1: <7.0 kPa), moderate (F2: 7.0 to <9.5 kPa), advanced (F3: 9.5 to <12.5 kPa), and cirrhosis (F4: ≥12.5 kPa) [10].

Clinical outcomes included deaths from all causes and the development of study-targeted diagnoses as defined in primary manuscript [5], which include liver, cardiac, renal diseases, HCV-related diagnoses, infections, and substance use. We also conducted analyses restricted to targeted liver diagnoses.

### Patient Consent

All participants provided informed written consent before enrollment and study procedures. The institutional review boards at each participating site approved the trial methods in concordance with ACTG procedures.

### Statistical Methods

For comparisons of binary or categorical measures between groups, Fisher exact tests were conducted. For comparisons of continuous measures between groups, Wilcoxon-Mann-Whitney rank-sum tests were conducted. The Spearman rank-order correlation coefficient was used to evaluate associations between fibrosis measures. For the changes in fibrosis scores over time, we took the difference in the score from the value at study entry at each follow-up visit and summarized the change at each follow-up visit. Cox proportional hazards models were developed to assess the effect of ELF on the time to clinical events (targeted diagnoses), adjusting for key demographic and health variables determined a priori. We also evaluated the clinical outcome that combined targeted diagnoses and death as a composite outcome as a supplemental analysis. The approach was similar to the primary analysis of clinical events conducted previously for the main study [5]. The start of follow-up time was defined as the time of VHICS entry in the Cox models where the ELF score at the time of VHICS entry was of interest; where the 26-week change in ELF score was of interest, the start of follow-up time was the week 26 visit when the ELF change could be assessed. The dichotomized ELF score (F3/F4, advanced vs <F3, nonadvanced) was used in the Cox models. The statistical test results should be interpreted with caution given the number of tests in this exploratory study about a novel biomarker, and the observed findings should be confirmed in additional studies. Analyses were conducted using SAS, version 9.4 (TS1M5, SAS/STAT 14.3; SAS Institute Inc., Cary, NC, USA).

## RESULTS

Data from 250 participants with any available ELF results were included in this analysis: 128 of 130 enrolled HCV/HIV participants; 122 of 125 enrolled HCV participants. Table 1 shows the demographics at entry into VHICS by HIV status, which were similar to those reported in the main study [5]. They were predominantly male sex at birth, with more Black than White participants and significant drug and alcohol use. There were no differences in demographics between HCV and HCV/HIV participants. Time on VHICS was longer in HCV/HIV compared to HCV participants because of slower enrollment of participants without HIV and early closure of VHICS [5]. Most (93%) participants had HCV genotype 1, as previously reported [5].

**Table 1.** VHICS Entry Characteristics and Follow-up Time

Characteristic (Q1, Q3)^a^ or (Percent)^a^	Total (N = 250)	With HIV (N = 128)	Without HIV (N = 122)
Median age, years	56 (50, 61)	53 (48, 58)	59 (53, 63)
Male sex at birth	193 (77%)	103 (80%)	90 (74%)
Race/ethnicity			
White, non-Hispanic	96 (38%)	44 (34%)	52 (43%)
Black, non-Hispanic	107 (43%)	56 (44%)	51 (42%)
Hispanic, any race	35 (14%)	25 (20%)	10 (8%)
Other	12 (5%)	3 (2%)	9 (7%)
Injection drug use			
Never	136 (54%)	73 (57%)	63 (52%)
Currently	1 (0%)	0 (0%)	1 (1%)
Previously	113 (45%)	55 (43%)	58 (48%)
Ever used drugs	185 (80%)	88 (77%)	97 (84%)
Missing	20	13	7
Ever smoked cigarettes	181 (76%)	91 (76%)	90 (77%)
Missing	13	8	5
Drank alcohol in the last 30 d	104 (44%)	56 (47%)	48 (41%)
Missing	14	8	6
Median BMI, kg/m²	27.6 (24.0, 30.9)	27.52 (24.2, 31.0)	27.71 (23.9, 30.7)
≥30.00	76 (31%)	38 (31%)	38 (32%)
Missing	8	5	3
Diabetes^b^	39 (16%)	18 (14%)	21 (17%)
Hyperlipidemia^b^	46 (18%)	27 (21%)	19 (16%)
Median weeks since DAA treatment	30 (40, 22)	37 (39, 24)	29(44, 20)
Median weeks of VHICS follow-up	215 (164, 239)	234 (181, 249)	189 (162, 218)

^a^Calculation based on the number with available data.

^b^Reported diagnosis or on medication indicated for this diagnosis.

Laboratory values prior to DAA therapy and at VHICS entry, within 1 year of achieving SVR are shown in Table 2. At VHICS entry, ALT and AST, APRI and FIB-4 values were lower than pre-DAA values. The proportions of HCV/HIV versus HCV participants with serum aminotransferase levels >upper limit of normal post-SVR were small (11% vs 6% for AST and 7% vs 2% for ALT). Although 16% and 18% reported diabetes and hyperlipidemia respectively on entry (Table 1), 38% had HOMA-IR > 3, with a higher proportion in HCV/HIV compared to HCV (*P* = .044). HCV RNA was <LLQ in all, confirming SVR status. Both APRI and FIB-4 decreased over time predominantly in the first year after DAA therapy. APRI was significantly higher in HCV/HIV versus HCV participants (*P* = .029) at VHICS entry but lower than values pre-DAA therapy.

**Table 2.** Laboratory Tests at the Time of HCV DAA Treatment Initiation Prior to VHICS Entry and at the Time of VHICS Entry

	At HCV DAA Treatment Initiation Median (Q1, Q3) or Number (Percent)				At VHICS Entry Median (Q1, Q3) or Number (Percent)			
Test	N^a^	Total	With HIV	Without HIV	N^a^	Total	With HIV	Without HIV
AST, mU/mL	239	41 (30, 65)	44 (32, 65)	41 (30, 63)	249	21 (18, 27)	23 (19, 29)	20 (17, 24)
>ULN		143 (60%)	78 (63%)	65 (58%)		21 (8%)	14 (11%)	7 (6%)
ALT, mU/mL	238	44 (29, 77)	47 (28, 79)	44 (30, 76)	249	17 (13, 23)	18 (14, 25)	16 (12, 22)
>ULN		120 (51%)	68 (55%)	52 (46%)		12 (5%)	9 (7%)	3 (2%)
Total bilirubin, mg/dL	234	0.50 (0.40, 0.80)	0.50 (0.30, 0.76)	0.59 (0.40, 0.80)	245	0.50 (0.40, 0.70)	0.50 (0.30, 0.70)	0.50 (0.40, 0.80)
Creatinine, mg/dL	236	0.97 (0.83, 1.10)	0.97 (0.85, 1.12)	0.95 (0.81, 1.10)	246	0.94 (0.61, 1.10)	0.96 (0.83, 1.11)	0.93 (0.79, 1.09)
INR	212	1.00 (0.95, 1.10)	1.00 (0.90, 1.10)	1.00 (1.00, 1.10)	239	1.00 (1.00, 1.10)	1.00 (1.00, 1.10)	1.00 (1.00, 1.10)
Platelets, ×10^3^ mm^3^	232	207 (163, 249)	203 (154, 247)	217 (170, 249)	250	203 (163, 246)	200 (160, 239)	209 (164, 251)
HCV RNA, log_10_ IU/mL	243	6.35 (5.85, 6.81)	6.41 (5.94, 6.96)	6.29 (5.79, 6.62)	246	<LLQ	<LLQ	<LLQ
HIV RNA								
<400 copies/mL	126	NA	125 (99%)	NA	122	NA	121 (99%)	NA
<LLQ		NA	113 (90%)	NA		NA	116 (95%)	NA
CD4, cells/mm^3^	125	NA	628 (457, 847)	NA	113	NA	700 (483, 890)	NA
HOMA-IR		NA	NA	NA	198	2.52 (1.53, 4.18)	2.80 (1.80, 4.73)	2.20 (1.51, 3.64)
>3						76 (38%)	40 (45%)	36 (33%)
APRI	216	0.56 (0.37, 1.03)	0.61 (0.38, 1.08)	0.50 (0.36, 0.89)	246	0.28 (0.21, 0.40)	0.30 (0.23, 0.43)	0.25 (0.19, 0.38)
>1.5		35 (16%)	19 (17%)	16 (16%)		2 (1%)	1 (1%)	1 (1%)
FIB-4	214	1.69 (1.13, 2.56)	1.69 (1.12, 2.64)	1.70 (1.15, 2.42)	246	1.33 (1.03, 1.96)	1.34 (1.03, 1.97)	1.33 (1.03, 1.91)
>3.25		30 (14%)	16 (14%)	14 (14%)		16 (7%)	7 (6%)	9 (7%)
ELF		NA	NA	NA	248	9.14 (8.59, 9.73)	8.99 (8.53, 9.55)	9.18 (8.63, 9.78)
≥9.8						52 (21%)	23 (18%)	29 (24%)

Abbreviation: NA, not applicable.

^a^Number with data for the test.

ELF scores were not available before entry into VHICS. At entry, more participants had advanced fibrosis by ELF score than FIB-4 or APRI: 52 participants compared to 16 participants by FIB-4 and 2 participants by APRI. There was no significant difference between HCV/HIV and HCV groups.

### Fibrosis Scores Over Time

**Figure 1.**
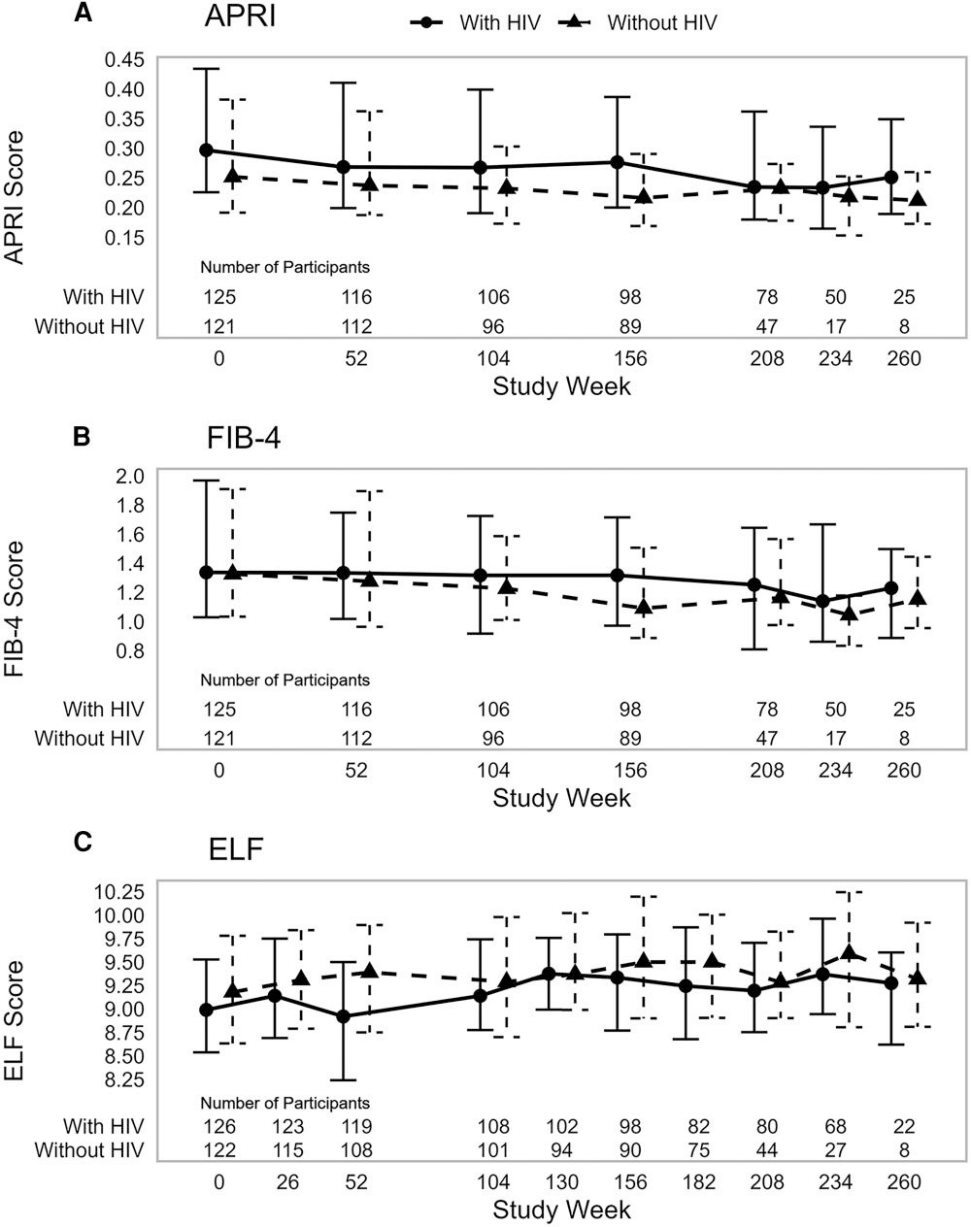
Depicts median (Q1, Q3) for APRI (*A*), FIB-4 (*B*), and ELF (*C*) at VHICS entry (week 0) and subsequent study weeks in HCV/HIV (solid lines with circles) and HCV (dotted lines with triangles) participants, including the number contributing data per study week.

Figure 1 depicts fibrosis scores over time for APRI, FIB-4, and ELF. Over time in VHICS, the median decreases for APRI and FIB-4 were similar between HCV/HIV and HCV participants. By week 260, the overall median (Q1, Q3) change from VHICS entry was modest at −0.08 (−0.19, −0.01) for APRI and −0.26 (−0.58, −0.03) for FIB-4, neither considered clinically significant per predefined criteria [9] and without meaningful differences between HCV/HIV and HCV. The proportions of participants in predefined ELF categories were similar between the 2 groups at entry and over time. Median changes from VHICS entry were similar in the 2 groups, with no clinically significant changes per predefined criteria. Week 52 changes from VHICS entry were least similar, with median (Q1, Q3) change of −0.03 (−0.55, 0.37) in HCV/HIV and median of −0.11 (−0.23, 0.51) in HCV, but still not clinically significant per predefined criteria [9]. The percentage of participants with increased ELF score at week 52 compared to VHICS entry was 49% in HCV/HIV and 57% in HCV. At other follow-up visits, at least 50% of the participants in each group had a higher ELF score compared to VHICS entry but were similar in the 2 groups. The proportion of participants with advanced fibrosis by ELF (F3/F4) increased from entry during the study follow-up and remained higher in HCV than HCV/HIV at each point. There were insufficient VCTEs to evaluate for fibrosis over time (20% with results after 1 year on VHICS and 30%–47% at years 2–4). Only 1 VCTE was done in 38 participants and 71 participants had 3 VCTE results available over time (not necessarily on consecutively scheduled years). In this small subset, the scores were stable over time (data not shown), consistent with the findings on APRI, FIB-4, and ELF.

Associations between ELF score and other measures of fibrosis showed weak positive correlations between ELF and APRI and between ELF and FIB-4 over time in VHICS. The correlation between ELF and APRI at week 0 was *r* = 0.378 (*P* < .001) in HCV/HIV and *r* = 0.417 (*P* < .001) in HCV. The correlation was *r* = 0.377 (*P* < .001) when the data from both groups were combined. The correlation between ELF and FIB-4 at week 0 was *r* = 0.449 (*P* < .001) in HCV/HIV, *r* = 0.490 (*P* < .001) in HCV, and *r* = 0.468 (*P* < .001), when combined. The ELF correlation estimates with both APRI and FIB-4 remained positive at subsequent study visits, but with a trend toward decreased significance as the numbers with available results declined.

### Associations Between ELF Score and Clinical Outcomes

There were 44 participants with clinical events (defined in the primary manuscript [5]), 29 among HCV/HIV (23%), and 15 (12%) among HCV participants. A participant could have more than 1 event. There was a lower cumulative incidence of study-targeted diagnoses in HCV compared to HCV/HIV, similar to what was reported in the A5320 primary manuscript [5]. Among the 44 participants who developed targeted diagnoses after VHICS entry, 70% (31/44) had ELF scores below F3 fibrosis stage (<9.8) at VHICS entry. Multivariable Cox proportional hazards regression models were used to assess the effect of week 0 ELF score (categorized as F0-2 vs F3/4) on the targeted diagnoses after VHICS entry, adjusting for HIV status, sex at birth, age (≤55 vs >55 years), race (White vs non-White), and body mass index (BMI; ≥30 vs <30 kg/m^2^) at VHICS entry. Week 0 and week 26 changes in ELF scores were not associated with time to first targeted diagnosis in the multivariable Cox model (data not shown). The hazard of developing targeted diagnoses was 1.95 times higher in HCV/HIV compared to HCV participants (HR [95% CI]: 1.951 [.999-3.810], *P* = .050), which was noted in the main study [5]. There was a trend that week 0 ELF < 9.8 was associated with lower risk of developing targeted diagnoses (0.559 [.277-1.128], *P* = .104).

**Figure 2.**
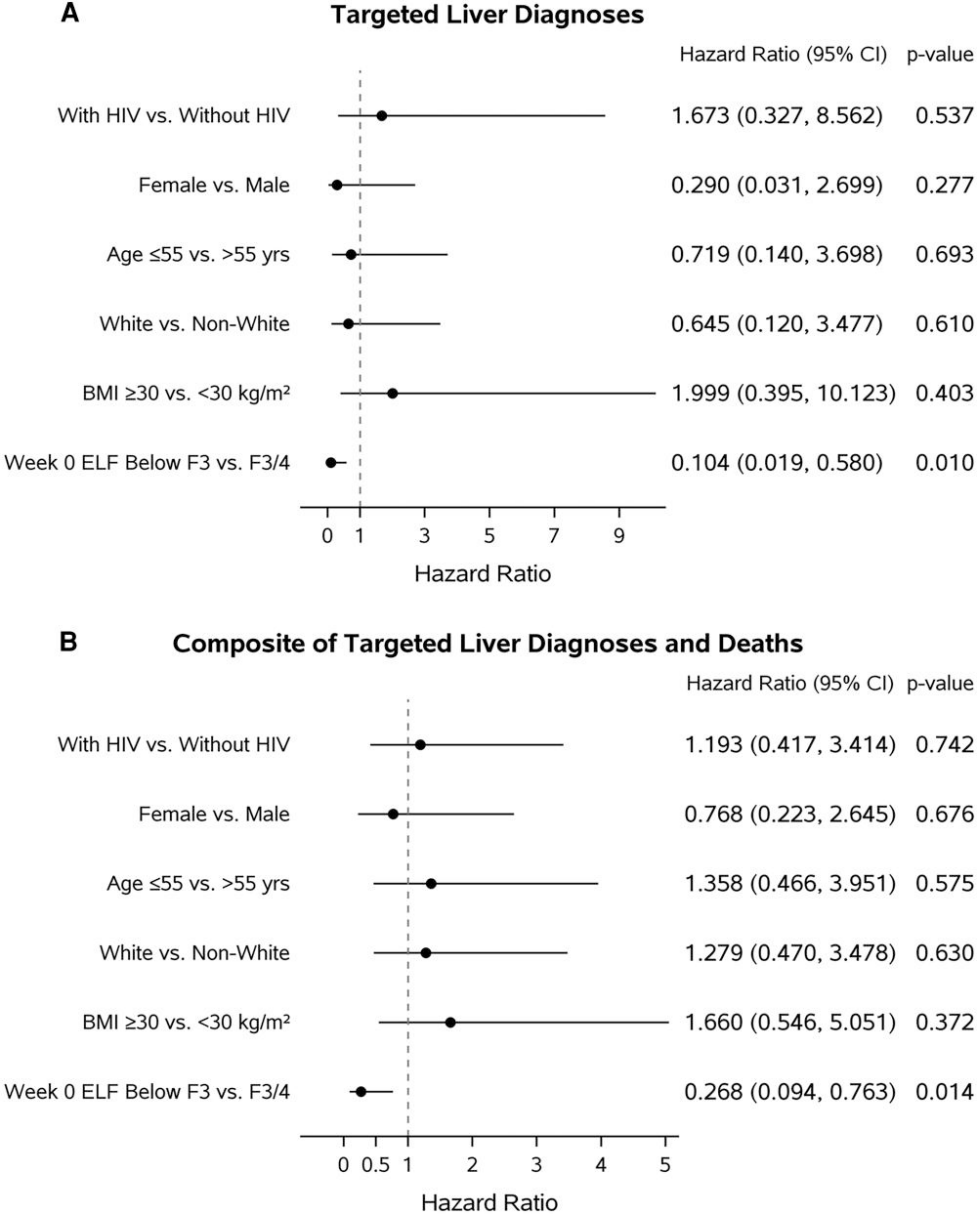
Hazard ratios with 95% confidence intervals from multivariate proportional hazards models for (*A*) targeted liver diagnoses and (*B*) composite of targeted liver diagnoses and all-cause deaths. The x-axis scales are different in (*A*) and (*B*). The model is based on 240 observations.

Within targeted diagnoses, there were 7 participants with hepatobiliary disorders or liver cancer: 4 hepatic disorders in HCV/HIV and 3 HCV with liver cancer. Among the 7 with targeted liver diagnosis or liver cancer, 5 had advanced fibrosis by ELF scores ≥9.8 at VHICS entry. The 2 HCV/HIV participants with ELF <9.8 had hepatic steatosis and steatohepatitis. ELF score below F3 fibrosis (ELF < 9.8) was associated with a lower chance of developing a targeted liver diagnosis compared to F3-F4 fibrosis (HR [95% CI]: 0.104 [.019-.580], *P* = .010) in the multivariable Cox regression model (Figure 2*A*). There was no difference between HCV/HIV and HCV groups when restricted to liver disease.

There were 9 deaths of any cause, 5 in HCV/HIV (observed at weeks 30, 77, 91, 108, 139) and 4 in the HCV (observed at weeks 42, 92, 141, 151) group. We also assessed development of clinical outcome that combined death and targeted diagnoses as a composite endpoint. A total of 49 participants (31 HCV/HIV and 18 HCV) were diagnosed with at least 1 of the targeted diagnoses or died after the VHICS entry. The composite that included deaths yielded similar multivariable Cox regression results. Restricting to liver diagnoses and combining with all-cause mortality, the multivariable Cox regression model revealed significant association between week 0 ELF and time to targeted liver diagnoses or death (Figure 2*B*, HR (95% CI): 0.268 (.094-.763), *P* = .014).

Given the significant association between week 0 ELF and time to targeted liver diagnoses or death, the participant characteristics at VHICS entry by ELF score (with or without advanced fibrosis) are presented in a supplementary table. Those with advanced fibrosis were older and more had evidence of metabolic disease with higher BMI with more diabetes. Laboratory testing showed that those participants with advanced fibrosis had higher entry ALT, lower platelets, and more participants had elevated HOMA-IR.

## DISCUSSION

A5320/VHICS was an observational, prospective, 5-year follow-up study in HCV/HIV and HCV participants enrolled within 1 year of completion of DAA-based HCV treatment. This analysis of 250 participants (128 HCV/HIV and 122 HCV) who achieved SVR on DAA therapy, characterized and compared long-term fibrosis assessments, using ELF, APRI, and FIB-4. APRI and FIB-4 decreased after HCV DAA therapy and APRI was higher in HCV/HIV compared to HCV participants (0.30 vs 0.25, *P* = .029). The proportion of participants with advanced fibrosis was higher by ELF scores than by APRI or FIB-4, at both study entry and during follow up. Over time, APRI and FIB-4 decreased from VHICS entry and were similar between groups but the decrease did not reach predefined clinical significance. In contrast, ELF scores did not decrease over time suggesting that the changes in APRI and FIB-4 were mainly due to decrease in markers of inflammation.

Although fibrosis markers and VCTE have proved excellent means of assessing liver fibrosis in chronic HCV, fibrosis assessment has been more complicated post-SVR. This is especially true for indices relying on use of inflammatory markers of liver disease as SVR leads to decrease necroinflammation with subsequent decrease in APRI and FIB-4. Indirect serum markers of fibrosis are not reliable after SVR [11]. Even VCTE is affected by hepatic inflammation/changes in AST/ALT. Post-SVR VCTE has been used to assess fibrosis but there are few studies of serum markers and/or VCTE assessment of long-term outcomes [1, 12]. Cossiga et al studied severe fibrosis using ELF and VCTE after SVR [13] but only in the first year after SVR. They found a decrease in liver fibrosis using both methods compared to baseline and that both ELF and VCTE were significantly associated with portal hypertension at baseline, but not with varices and ascites. Our study differs from this smaller study in that fewer patients had advanced fibrosis; it commenced during the first year after SVR; we followed participants for up to 5 years. We found that ELF scores were associated with liver-related outcomes and death.

The ELF score does not rely on clinically available tests but on markers of fibrogenesis and matrix degradation and can be performed on stored serum or plasma. Studies of untreated patients with HCV have shown ELF predicts mortality in both HCV and HCV/HIV participants [4]. We found that ELF scores were associated with development of a liver-related diagnosis and death, which has been found in HCV and HCV/HIV patients before therapy [3, 4]. Liver cancer after SVR has been widely reported and was seen in our study; thus, it remains unclear when to stop hepatocellular cancer screening after SVR.

There were several limitations in this analysis. ELF was not available before VHICS entry, thus scores before DAA therapy were not available. The number of participants available for analysis of fibrosis scores decreased over time in VHICS (see Figure 1; from years 1 through 5: 92%, 82%, 76%, 51%, and 13%, respectively) but was similar between groups. Time from DAA end of treatment to VHICS entry differed between groups with a median of 29 weeks for HCV/HIV SVR and 37 weeks for HCV SVR. The effect of metabolic dysfunction-associated steatotic liver disease was captured as targeted clinical events but not otherwise evaluated. There is an association between direct biomarkers of liver fibrosis such as ELF and indirect markers in a number of other chronic liver diseases; it is well described in metabolic-dysfunction associated steatohepatitis [14].

The supplementary table highlighted that at VHICS entry, metabolic risk factors for liver disease appeared higher in those with evidence of advanced fibrosis. This warrants further study as steatotic liver disease and metabolic risk factors are common in chronic HCV and more prevalent in people with HIV [15]. VCTE were only available in 20% at entry and 30% to 47% at years 2 through 4. We conducted a number of statistical tests in this exploratory study to not miss any potentially significant finding, and further studies on ELF can confirm our findings. We also note that there may be additional covariates that are important to consider in the model to assess the effect of ELF on the development of clinical events; we included what we considered to be key that were in line with our primary paper. Furthermore, our data, with a limited number of clinical events, may not have sufficient power to detect a small effect.

In summary, although “indirect” fibrosis tests have been reported to decrease after SVR, this appears to be mainly due to decreases in inflammation. ELF showed minimal change after SVR suggesting persisting fibrosis; this is further supported by continued occurrence of liver-related clinical events. ELF was better at determining severe fibrosis/cirrhosis and higher ELF at entry into VHICS was associated with liver-related clinical events and death. Where VCTE is not readily available, ELF could be measured early after SVR to predict liver-related outcomes and death and may be preferrable to FIB-4, APRI, or even VCTE because of the lack of interference from the resolution of inflammation. Further studies after SVR should include longer-term follow-up to monitor for clinical events and consider ELF measurements to assess persisting fibrosis.

## Supplementary Material

ofaf804_Supplementary_Data

## Supplementary Data

Supplementary materials are available at *Open Forum Infectious Diseases* online. Consisting of data provided by the authors to benefit the reader, the posted materials are not copyedited and are the sole responsibility of the authors, so questions or comments should be addressed to the corresponding author.
