# Supplementary material for: Evaluation of Liver Fibrosis Change After DAA-induced Cure of Hepatitis C in Participants With and Without HIV: ACTG A5320 Viral Hepatitis C Infection Long-term Cohort Study (VHICS)
Source: Open Forum Infect Dis. 2026 Jan 14;13(1):ofaf804. doi: 10.1093/ofid/ofaf804 (PMC12803019; doi:10.1093/ofid/ofaf804)
Supplement: ofaf804_Supplementary_Data [file ofaf804_supplementary_data.docx]

**Table S1: V-HICS Entry Characteristics by ELF Score**

| **Characteristic**  **Median (Q1, Q3)^a^ or (Percent)^a^** | **Total (N=248)** | **F0-F2 (N=196)** | **F3-F4 (N=52)** |
| --- | --- | --- | --- |
| Age (years) | 56 (50, 61) | 55 (49, 60) | 58.5 (53.0, 62.0) |
| Sex at birth | 191 (77%) | 157 (80%) | 34 (65%) |
| Race/Ethnicity |  |  |  |
| White, Non-Hispanic | 96 (39%) | 79 (40%) | 17 (33%) |
| Black, Non-Hispanic | 106 (43%) | 81 (41%) | 25 (48%) |
| Hispanic, Any Race | 35 (14%) | 26 (13%) | 9 (17%) |
| Other | 11 (4%) | 10 (5%) | 1 (2%) |
| Injection drug use |  |  |  |
| Never | 134 (54%) | 111 (57%) | 23 (44%) |
| Currently | 1 (0%) | 1 (1%) | 0 (0%) |
| Previously | 113 (46%) | 84 (43%) | 29 (56%) |
| Ever used drugs | 185 (80%) | 140 (78%) | 45 (88%) |
| Missing | 18 | 17 | 1 |
| Ever smoked cigarettes | 180 (76%) | 139 (75%) | 41 (80%) |
| Missing | 12 | 11 | 1 |
| Drank alcohol in the last 30 days | 103 (44%) | 86 (47%) | 17 (33%) |
| Missing | 13 | 12 | 1 |
| BMI (kg/m2) | 27.6 (24.0, 30.9) | 26.7 (23.9, 30.0) | 30.5 (27.6, 34.0) |
| ≥ 30 | 76 (32%) | 47 (25%) | 29 (58%) |
| Missing | 8 | 6 | 2 |
| Diabetes^b^ | 39 (16%) | 23 (12%) | 16 (31%) |
| Hyperlipidemia^b^ | 45 (18%) | 37 (19%) | 8 (15%) |
| Weeks since DAA treatment | -29.9 (-39.3, -21.9) | -30.4 (-40.1, -23.9) | -26.5 (-35.9, -18.1) |
| AST (mU/mL) | 21.0 (17.9, 27.0) | 20 (17, 25) | 26.0 (20.5, 30.0) |
| > ULN | 21 (9%) | 11 (6%) | 10 (19%) |
| Missing | 1 | 1 | 0 |
| ALT (mU/mL) | 17 (13, 23) | 16 (13, 23) | 18.5 (14.0, 26.5) |
| > ULN | 12 (5%) | 7 (4%) | 5 (10%) |
| Missing | 1 | 1 | 0 |
| Total Bilirubin (mg/dL) | 0.5 (0.4, 0.7) | 0.5 (0.4, 0.7) | 0.6 (0.3, 0.9) |
| Missing | 5 | 3 | 2 |
| Serum Creatinine (mg/dL) | 0.9 (0.8, 1.1) | 1.0 (0.8, 1.1) | 0.9 (0.8, 1.2) |
| Missing | 4 | 4 | 0 |
| INR (ratio) | 1.0 (1.0, 1.1) | 1.0 (1.0, 1.1) | 1.1 (1.0, 1.1) |
| Missing | 11 | 9 | 2 |
| Platelets (x10³/mm³) | 203.0 (163.5, 246.5) | 208.5 (174.5, 252.7) | 158.0 (119.0, 212.5) |
| HOMA-IR | 2.5 (1.5, 4.2) | 2.3 (1.5, 3.5) | 4.1 (2.2, 6.9) |
| >3 | 75 (38%) | 49 (32%) | 26 (59%) |
| Missing | 51 | 43 | 8 |
| APRI | 0.3 (0.2, 0.4) | 0.3 (0.2, 0.4) | 0.4 (0.3, 0.7) |
| > 1.5 | 2 (1%) | 0 (0%) | 2 (4%) |
| Missing | 4 | 4 | 0 |
| FIB-4 | 1.3 (1.0, 2.0) | 1.3 (1.0, 1.8) | 2.0 (1.4, 3.0) |
| > 3.25 | 16 (7%) | 4 (2%) | 12 (23%) |
| Missing | 4 | 4 | 0 |
| HIV-1 RNA (c/mL)^c^ |  |  |  |
| <400 | 119 (99%) | 97 (99%) | 22 (100%) |
| Missing | 6 | 5 | 1 |
| CD4 (cells/mm^3^) | 703 (483, 890) | 708 (521, 902) | 558 (450, 868) |
| Missing^c^ | 15 | 13 | 2 |

^a^ Calculation based on the number with available data

^b^ Reported diagnosis or on medication indicated for this diagnosis

^c^ HCV/HIV participants only: 126 (103 with F0-F2 and 23 with F3-F4) with Week 0 ELF data
